# Enhancement of Tendon–Bone Healing for Anterior Cruciate Ligament (ACL) Reconstruction Using Bone Marrow-Derived Mesenchymal Stem Cells Infected with BMP-2

**DOI:** 10.3390/ijms131013605

**Published:** 2012-10-22

**Authors:** Yu Dong, Qingguo Zhang, Yunxia Li, Jia Jiang, Shiyi Chen

**Affiliations:** 1 Department of Sports Medicine, Huashan Hospital, No. 12, Wulumuqi Zhong Road, Shanghai 200040, China; E-Mails: dongyu.dy@163.com (Y.D.); liyunxia912@yahoo.com.cn (Y.L.); jessicajj19@hotmail.com (J.J.); 2 Department of Orthopedics, Taizhou Hospital, Wenzhou Medical College, Linhai, Zhejiang 317000, China; E-Mail: xingzhe7@126.com

**Keywords:** tendon-bone healing, anterior cruciate ligament (ACL), reconstruction, bone marrow-derived mesenchymal stem cells

## Abstract

At present, due to the growing attention focused on the issue of tendon–bone healing, we carried out an animal study of the use of genetic intervention combined with cell transplantation for the promotion of this process. Here, the efficacy of bone marrow stromal cells infected with bone morphogenetic protein-2 (BMP-2) on tendon–bone healing was determined. A eukaryotic expression vector containing the BMP-2 gene was constructed and bone marrow-derived mesenchymal stem cells (bMSCs) were infected with a lentivirus. Next, we examined the viability of the infected cells and the mRNA and protein levels of BMP-2-infected bMSCs. Gastrocnemius tendons, gastrocnemius tendons wrapped by bMSCs infected with the control virus (bMSCs+Lv-Control), and gastrocnemius tendons wrapped by bMSCs infected with the recombinant BMP-2 virus (bMSCs+Lv-BMP-2) were used to reconstruct the anterior cruciate ligament (ACL) in New Zealand white rabbits. Specimens from each group were harvested four and eight weeks postoperatively and evaluated using biomechanical and histological methods. The bMSCs were infected with the lentivirus at an efficiency close to 100%. The BMP-2 mRNA and protein levels in bMSCs were significantly increased after lentiviral infection. The bMSCs and BMP-2-infected bMSCs on the gastrocnemius tendon improved the biomechanical properties of the graft in the bone tunnel; specifically, bMSCs infected with BMP-2 had a positive effect on tendon–bone healing. In the four-week and eight-week groups, bMSCs+Lv-BMP-2 group exhibited significantly higher maximum loads of 29.3 ± 7.4 N and 45.5 ± 11.9 N, respectively, compared with the control group (19.9 ± 6.4 N and 21.9 ± 4.9 N) (*P* = 0.041 and *P* = 0.001, respectively). In the eight-week groups, the stiffness of the bMSCs+Lv-BMP-2 group (32.5 ± 7.3) was significantly higher than that of the bMSCs+Lv-Control group (22.8 ± 7.4) or control groups (12.4 ± 6.0) (*p* = 0.036 and 0.001, respectively). Based on the histological findings, there was an increased amount of perpendicular collagen fibers formed between the tendon and bone in the bMSCs+Lv-Control and bMSCs+Lv-BMP-2 group, compared with the gastrocnemius tendons. The proliferation of cartilage-like cells and the formation of fibrocartilage-like tissue were highest within the bone tunnels in the bMSCs+Lv-BMP-2 group. These results suggest that this lentivirus can be used to efficiently infect bMSCs with BMP-2. Furthermore, tendons wrapped by bMSCs+Lv-BMP-2 improved tendon–bone healing.

## 1. Introduction

The anterior cruciate ligament (ACL) is an important structure in the knee and plays a vital role in maintaining knee stability [1–3]. Damage to the anterior cruciate ligament (ACL) is a common and serious sports injury. The success or failure of ACL injury reconstruction is determined by the degree of knee function recovery and thus determines the degree of the player’s athletic ability recovery and athletic performance, and even the length of the sports career [4,5]. Improper treatment could cause knee instability, movement dysfunction, intra-articular meniscus and cartilage damage, and ultimately lead to osteoarthritis of the knee [1,2]. Surgical reconstruction using soft-tissue autografts, including hamstring tendon and the patellar-tendon bone, has been the standard of care for a torn ACL. Generally, these autografts can restore initial knee stability, but a number of associated issues remain. One-quarter of patients have less than satisfactory long-term results, with clinical studies demonstrating osteoarthritic changes in the knee [3–5]. Thus, there is a persistent need to develop better treatment regimens for improved patient outcome. The success of surgery largely depends on the biological healing between the graft and bone tunnel. Early connection of the tendon–bone interface is the weakest link. Therefore, it is important to determine how to stimulate the graft and bone healing for functional exercises, sports, and daily activities as early as possible.

Bone marrow-derived mesenchymal stem cells (bMSCs) are multipotent adult stem cells and have become an important source of cells for engineered tissue repair and cell therapy [6]. Their osteogenic differentiation potential has been characterized in several *in vitro* studies. In addition, small animal model-based studies have indicated their bone formation capability *in vivo* when implanted with biodegradable scaffolding, indicating great potential for therapeutic application [7,8].

Some studies suggest that the formation of bone tissue is a process of osteogenesis by a variety of growth factors participating in and regulating mechanical tension [9–12]. Bone morphogenetic protein (BMP) is the most potent growth factor with osteoinductive properties [13,14]. Of all BMPs, BMP-2 has the most well-known effect on promotion of the process of defect repair and fracture healing, and can induce pluripotent stem cells and bone progenitor cells to differentiate into osteogenic and chondrogenic cells [15–19].

Based on this background, we utilized cells genetically modified with BMP-2 to improve tendon–bone healing. The purpose of this study was to determine the efficacy of promoting tendon–bone healing after ACL reconstruction by bMSC transplantation. We hypothesized that the transplantation of genetically modified BMP-2 cells would significantly enhance the osseointegration of the ligament graft within the host bone.

## 2. Results and Discussion

### 2.1. *In Vitro* Studies

#### 2.1.1. Infection of bMSCs with Lentivirus

**Figure 1:**
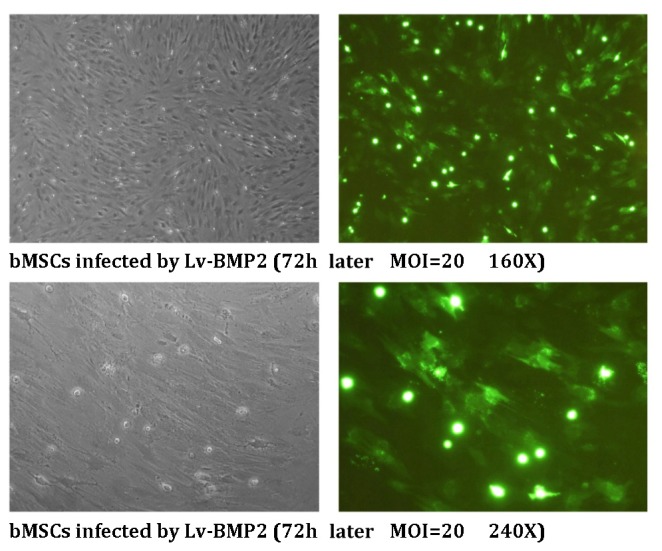
Efficiency of infection of bMSCs with lentivirus. During the exponential growth phase, bMSCs were infected with Lv-BMP2 at an MOI = 20. After 72 h, GFP expression was examined by fluorescence microscopy at the indicated magnifications.

The bMSCs were infected with the lentivirus at an MOI = 20. According to fluorescence microscopy visualization, the infection efficiency was ~100% at 72 h after viral infection (Figure 1).

#### 2.1.2. MTT Assay

**Figure 2:**
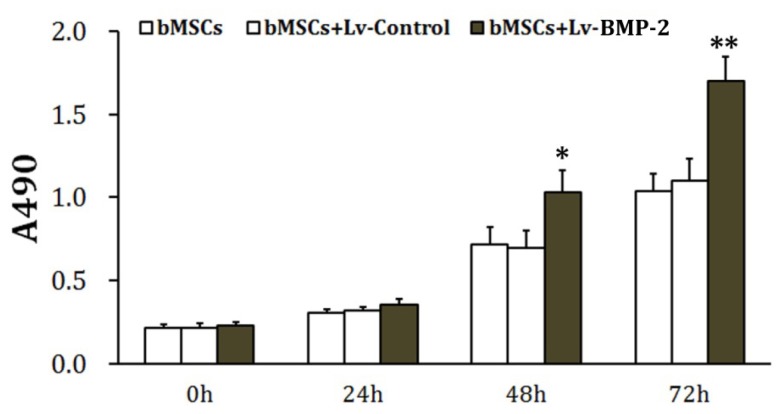
Determination of cell viability. During the exponential growth phase, bMSCs were infected with either the Lv-Control or Lv-BMP-2 at an MOI = 20 and seeded into 96-well plates at 1 × 10^5^/well 72 h later, followed by incubation under normal culture conditions. The MTT assay was performed as described in the Materials and Methods. The absorbance (A) at 490 nm was determined to estimate changes in cell viability. * *p* < 0.05 *vs.* bMSCs and ** *p* < 0.01 *vs*. bMSCs.

The bMSCs were infected with the control virus (Lv-Control) or the recombinant BMP-2 virus (Lv-BMP-2) at an MOI = 20. The cells were seeded and incubated under normal culture conditions 72 h after infection. The MTT assay was performed every 24 h. Results showed that 48 and 72 h later, the proliferative activity of BMP-2-overexpressing bMSCs was significantly increased compared to the control group (*p* < 0.05) (Figure 2). In contrast, there was no significant difference between the group infected with the Lv-Control and the control group (*p* > 0.05). These results indicate that lentiviral infection had no obvious detrimental effect and that overexpression of BMP-2 enhances the proliferative activity of bMSCs.

#### 2.1.3. Real-Time Polymerase Chain Reaction

**Figure 3:**
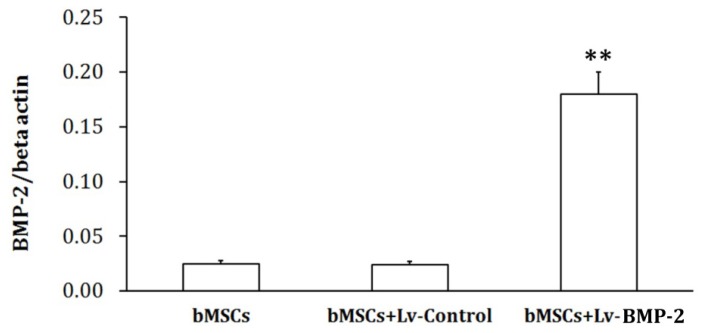
Determination of mRNA content. During the exponential growth phase, bMSCs were infected with either the Lv-Control or Lv-BMP-2 at an MOI = 20 and subjected to RNA extraction 72 h later. The RNA was transcribed into cDNA using reverse transcriptase and analyzed by real-time PCR. The results of relative quantification were analyzed by the ΔCt method. B-Actin served as the internal control. ** *p* < 0.01 *vs*. bMSCs.

The control virus (Lv-Control) and recombinant BMP-2 virus (Lv-BMP-2) were used to infect bMSCs at an MOI = 20. The cells were collected 72 h later, and total RNA was extracted using TRIzol reagent (Invitrogen, USA). Agarose gel electrophoresis revealed no significant DNA contamination in the RNA, while A260/A280 ratios were 1.8–2.0, indicating no significant degradation of or organic pollution in the RNA. Amplification curves from polymerase chain reaction (PCR) showed that the amplification efficiency of the PCR system was normal and the single melting curve indicated good primer specificity. These results showed that the BMP-2 mRNA level in mesenchymal cells was increased significantly 72 h after lentiviral infection compared to the control (*p* < 0.01), while there was no significant difference in the BMP-2 mRNA contents of the cells infected with the control virus and the control (*p* > 0.05) (Figure 3).

#### 2.1.4. Western Blot Analysis

**Figure 4:**
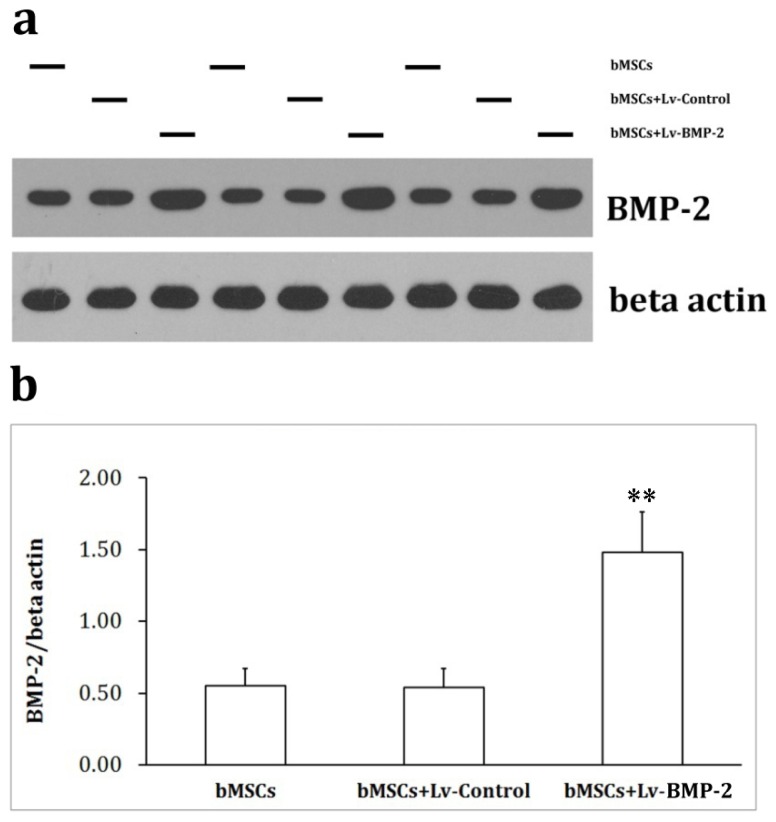
Protein contents determined by Western blotting. During the exponential growth phase, bMSCs were infected with either the Lv-Control or Lv-BMP-2 at an MOI = 20 and subjected to Western blotting 72 h later. Cell lysates were prepared, electrophoresed and immunoblotted for BMP-2 and β-actin (**a**) The optical densities of bands were analyzed by TotalLab and the relative quantification of BMP-2 was performed (**b**). ** *p* < 0.01 *vs*. bMSCs.

According to Western blot analysis, BMP-2 and β-actin showed molecular weights of 60 kDa and 43 kDa, respectively. The optical density analysis software, TotalLab, was used to analyze the optical density of bands. Results showed that the BMP-2 protein content in the virus-infected cells was significantly increased compared to the control group (*p* < 0.05), while there was no significant difference between BMP-2 protein levels of the cells infected with the control virus and the control group (*p* > 0.05) (Figure 4).

The junction between the transplanted tendon and the bone is a weak link in the early healing process [20–24]. Therefore, one of the focuses of the sports medicine field is how to effectively promote tendon and bone healing and how to strengthen the quality of healing. In recent years, scholars have made significant progress in the promotion of tendon–bone healing. Kohno *et al*. [25] reported that fibroblast growth factor 2 (FGF-2) and vascular endothelial growth factor (VEGF) promoted early fiber integration of tendon–bone healing after ACL reconstruction in a rabbit model. BMP-2 and BMP-7 significantly promoted the osseointegration of tendon and bone. Additionally, Huangfu *et al*. [26] showed that calcium phosphate, used for filling the bone tunnel, significantly improved tendon–bone healing after ACL reconstruction in beagles. Demirag *et al*. [27] found that α2-macroglobulin blockade of matrix metalloproteinases (MMPs) enhanced tendon–bone healing. This α2-macroglobulin effect may be mediated solely through its effect on a subset of MMPs that are present at the healing interface. Although these studies have made significant progress, the effectiveness and clinical applications of these results must still be investigated.

bMSCs are produced by the mesoderm and have multi-differentiation potential and strong self-proliferating activity [28]. BMP-2 can efficiently induce activity *in vivo*, and particularly an ectopic osteogenic activity. Therefore, we hypothesized that combination of the advantages of both bMSCs and BMP-2 might significantly promote tendon–bone healing [16,18]. The lentiviral vector system is the most widely-used of all present gene transfection vectors. In our study, we transfected the BMP-2 gene into bMSCs by lentiviral delivery. The bMSCs were infected with the lentivirus at an efficiency of ~100%. Lentiviral infection had no obvious detrimental effect, and overexpression of BMP-2 enhanced the proliferative activity of bMSCs. BMP-2 mRNA and protein levels in bMSCs were increased significantly 72 h after lentiviral infection compared to the control group (*p* < 0.05).

Recently, Lee *et al*. [29] further found that brief stimulation with BMP-12 *in vitro* is sufficient to induce rat bone marrow-derived mesenchymal stem cells (BM-MSC) differentiation into tenocytes, and that this phenotype is sustained *in vivo*. These results suggest that pretreating BM-MSCs with BMP-12 prior to *in vivo* transplantation may be useful in MSC-based tendon reconstruction or tissue engineering. Haddad-Weber *et al*. [30] described the *in vitro* potential of MSC and ACL cells as undergoing ligamentogenic differentiation upon transduction with adenoviral vectors encoding the human cDNA for BMP 12 and BMP 13. They found that both MSC and ACL fibroblasts are capable of ligamentogenic differentiation with these factors. This information may aid in the development of biologic approaches to repair and restore ACL after injury. These strategies may provide some potential ways for improving ACL renconstruction using BMP.

### 2.2. *In Vivo* Studies

#### 2.2.1. Gross Observation

A total of 60 ACL reconstructions with grafts were performed in 30 rabbits in this study. In the study, there were no premature deaths, nor were there any joint infections. Moreover, no evidence of any joint infection was noted on postmortem examination of the limbs. All of the rabbits were euthanized at the planned times.

#### 2.2.2. Mechanical Findings

**Figure 5:**
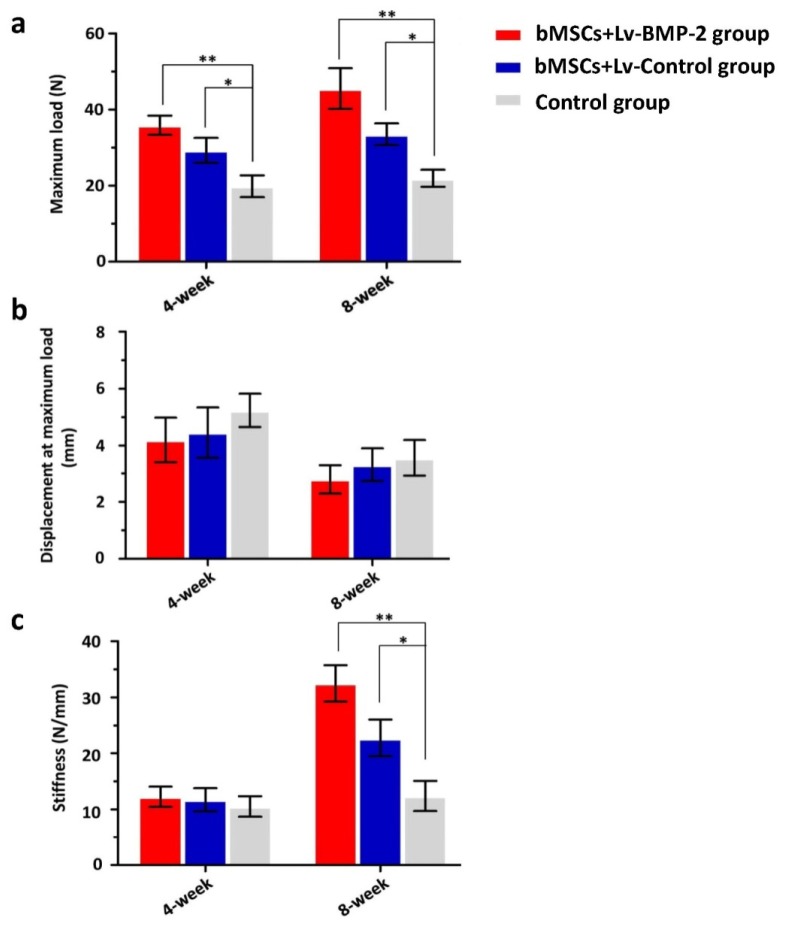
Mechanical examination of graft-bone healing in a rabbit model at various time points after surgery. (**a**) Maximal load; (**b**) Displacement at maximum load; (**c**) Stiffness. * LSD *t*-test, *p* < 0.01; ** LSD *t*-test, *p* < 0.05.

In the four-week groups, bMSCs+Lv-Control group and bMSCs+Lv-BMP-2 group exhibited significantly higher maximum loads of 35.9 ± 5.6 N and 29.3 ± 7.4 N, respectively, compared with the control group (19.9 ± 6.4 N) (*P* = 0.002 and *p* = 0.041, respectively; least significant difference [LSD] *t*-test) (Figure 5a). There was no significant difference in the maximum load between the bMSCs+Lv-Control group and bMSCs+Lv-BMP-2 group (*p* = 0.133; LSD *t*-test) (Figure 5a). The displacement at maximum load and stiffness did not differ significantly among the three groups (*p* = 0.973 and *p* = 0.811, respectively; one-way analysis of variance (ANOVA)) (Figure 5 b,c).

The eight-week data showed that the maximum load of the bMSCs+Lv-BMP-2 group (45.5 ± 11.9 N) was significantly higher than that of either the bMSCs+Lv-Control group (33.5 ± 6.4 N) or the control group (21.9 ± 4.9 N) (*p* = 0.042 and *p* = 0.001, respectively; LSD *t*-test) (Figure 5a). The maximum load of the bMSCs+Lv-Control group was higher than that of the control group (*p* = 0.048; LSD *t*-test) (Figure 5a). There was no statistical difference in the displacement at maximum load among the three groups (*p* = 0.647; one-way ANOVA) (Figure 5b). However, the stiffness of the bMSCs+Lv-BMP-2 group (32.5 ± 7.3) was significantly higher than that of the bMSCs+Lv-Control group (22.8 ± 7.4) or control group (12.4 ± 6.0) (*p* = 0.036 and *p* = 0.001, respectively; LSD *t*-test) (Figure 5c). Furthermore, the stiffness of the bMSCs was higher than that of the control group (*p* = 0.036; LSD *t*-test) (Figure 5c).

Lim *et al*. [31] reported that bilateral ACL reconstructions using hamstring tendon autografts were performed on adult rabbits. Grafts were coated with mesenchymal stem cells (MSCs) in a fibrin glue carrier in one limb, and fibrin glue only in the other. Assessment was done at two, four, and eight weeks. Biomechanically, there was no statistical difference between limbs at two and four weeks. At eight weeks, the MSC-enhanced grafts had significantly higher failure load and stiffness. Kanaya *et al*. [32] injected MSCs into intra-articular joint in partially torn anterior cruciate ligaments in a rat model. They found that the ultimate failure load of the femur-ACL-tibia complex in the MSC (+) group was significantly higher than that in the MSC (−) group at four weeks after surgery, which means the intra-articular injection of mesenchymal stromal cells can be a viable option for treating partially torn knee ACLs. In our study, we found that bMSCs+Lv-Control on the gastrocnemius tendon improved the biomechanical properties of the graft in the bone tunnel. With regard to the four-week groups, bMSCs+Lv-Control group exhibited a significantly higher maximum load than did the control group (*p* = 0.002). In the eight-week groups, the maximum load in the bMSCs+Lv-Control group was higher than that in the control group (*p* = 0.048). Furthermore, the stiffness of the bMSCs+Lv-Control group was also higher than that of the control group (*p* = 0.036). These results suggest that bMSCs could enhance tendon–bone healing of ACL reconstructions.

Kohno *et al*. [25] reported that BMP-2 was detected throughout the 12-week study period and was present at high concentrations near the bone. Rodeo *et al*. [33] showed that BMP-2 improved the formation of new bone and fibrocartilage at the healing tendon attachment site, resulting in improved load to failure. In addition, recombinant human BMP-2 (rhBMP-2) exerted a strong, positive, dose-dependent effect on osseointegration at the tendon-bone junction. No tunnel widening was detected with rhBMP-2 using the calcium phosphate carrier [34]. We processed ACLs reconstructed with a gastrocnemius tendon wrapped by bMSCs, which were infected with the Lv-BMP-2 and immobilized in fibrin glue. Finally, we found that in the four-week groups, bMSCs+Lv-Control and bMSCs+Lv-BMP-2 groups exhibited significantly higher maximum loads, respectively, compared with the control group (*p* = 0.002 and *p* = 0.041). There was no significant difference in the maximum load between the bMSCs+Lv-Control and bMSCs+Lv-BMP-2 group (*p* = 0.133). The displacement at maximum load and stiffness did not differ significantly among the three groups (*p* = 0.973 and *p* = 0.811, respectively). The eight-week data showed that the maximum load of the bMSCs+Lv-BMP-2 group was significantly higher than that of either the bMSCs+Lv-Control or the control group (*p* = 0.042 and *p* = 0.001, respectively). However, the stiffness of the bMSCs+Lv-BMP-2 group was significantly higher than that of the bMSCs+Lv-Control or control group (*p* = 0.036 and *p* = 0.001, respectively). These results indicate that genetic intervention combined with cell transplantation promotes tendon–bone healing.

#### 2.2.3. Histological Findings

Four weeks after surgery, in the control group, 40% of limbs showed that tendon–bone healing began with the formation of fibrovascular interface tissue, while 60% showed no fibrovascular interface tissue. In contrast, no fibrovascular interface tissue was found either in bMSCs+Lv-Control group or in bMSCs+Lv-BMP-2 group.

**Figure 6:**
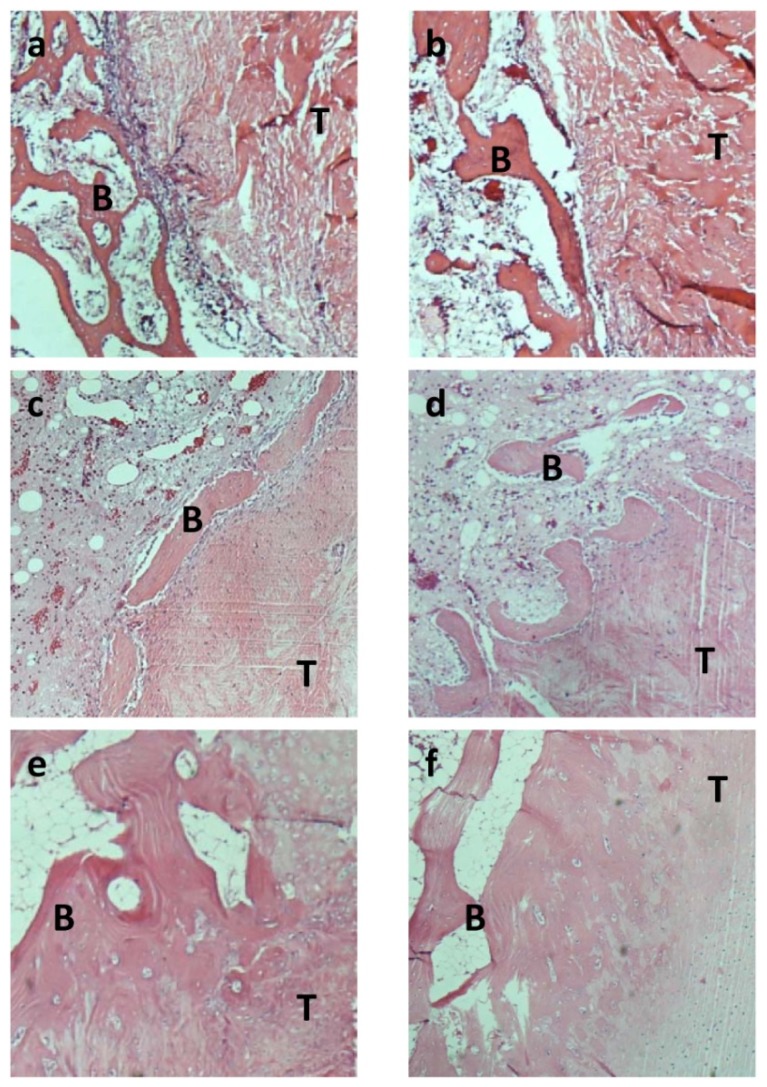
Haematoxylin and eosin (H & E) staining of tendon–bone at four and eight weeks after surgery. This figure shows the histology of the tendon (**T**)-bone (**B**) interface among the control group, bMSCs and bMSCs infected with BMP-2 either four (**a**, **c** and **e**) or eight (**b**, **d** and **f**) weeks after surgery. Fibrous tissue was observed eight weeks after surgery in the control group (**b**). However, chondrocytes and a greater number of perpendicular fibers were observed at the interface between tendon and bone (magnification 100×) in the bMSCs (**c** and **d**) and BMP-2-transfected bMSCs (**e** and **f**) groups.

Eight weeks after surgery, in the control group, most of the fibrous tissue was parallel to the load axis (80%), but some direct collagen fibers were observed in two limbs (Figure 6a,b). However, in bMSCs+Lv-Control and bMSCs+Lv-BMP-2 group, more perpendicular collagen fibers between the tendon and bone were observed (60% and 80%, respectively) (Figure 6c–f). Furthermore, the proliferation of cartilage-like cells within the bone tunnels were higher in the bMSCs+Lv-BMP-2 group, compared with the bMSCs+Lv-Control and control groups (60%, 20%, 0%, respectively). Besides, the formation of the fibrocartilage-like tissue were also higher in the bMSCs+Lv-BMP-2 group than other two groups (80%, 20%, 20%, respectively).

Healing of a tendon graft in a bone tunnel depends on bone ingrowth into the interface between tendon and bone. Lim *et al*. [31] found that control reconstructions only using hamstring tendon autograft showed mature scar tissue with some Sharpey’s-like fibers spanning the tendon–bone interface at eight weeks after operation. The MSC-enhanced reconstructions had large areas of cartilage cells at the tendon–bone junction at two weeks. A mature zone of cartilage was seen gradually blending from bone into the tendon grafts by eight weeks, and this zone stained strongly for type II collagen and showed histologic characteristics similar to normal rabbit ACL insertions. Kanaya *et al*. [32] found that in the MSC (−) group, the transected area retracted with increasing time, and the gap remained void of any tissues at all time points after surgery. In the MSC (+) group at two and four weeks after surgery, the transected area was covered with healing tissues in which GFP-positive cells were detected. Furthermore, the histologic score of the MSC (+) group was significantly better than that of the MSC (−) group.

A study by Hong *et al.* [35] showed that a bone tunnel with increased bMSCs may improve the insertion healing of tendon to bone in a rabbit model through the formation of perpendicular collagen fibers and increased proliferation of cartilage-like cells at four weeks. In our study, histological results indicated formation of more perpendicular collagen fibers between the tendon and bone in the bMSCs+Lv-Control and bMSCs+Lv-BMP-2 group compared to the control group. The proliferation of cartilage-like cells and the formation of fibrocartilage-like tissue within the bone tunnels in the bMSCs+Lv-BMP-2 group were higher than in the other two groups, which is similar to data reported elsewhere [35].

Some clinical studies have shown that the effect of ACL reconstruction is influenced by the extent of healing between the tendon transplanted and the bone after surgery [20–24]. Our study showed that tendons wrapped by bMSCs+Lv-BMP-2 had the potential to improve tendon–bone healing, which may be a novel method offering the potential for more physiologic and biomechanically stronger ligament reconstructions in clinical practice. However, more animals and clinical experiments will be carried out to confirm the effectiveness.

However, there are several limitations of this study. First, the small number of experimental animals is not enough to avoid selection bias. Second, we just observed two time points (four weeks and eight weeks after surgery), instead of the longer observation time, because the original intention of the experiment was to detect the early healing of tendon grafts. Third, when tendons were wrapped by fibrin glue solution, including autologous bMSCs+Lv-Control or bMSCs+Lv-BMP-2, these coatings might not be well distributed on the treatment limbs. In any case, in the subsequent experiment by our group, we would try to settle the aforementioned limitations.

## 3. Experimental Section

### 3.1. *In Vitro* Studies

#### 3.1.1. Cell Culture

bMSCs were isolated from the tibia of four healthy, mature New Zealand white rabbits (average weight, 3.0 kg; purchased from the Experimental Animal Department of Shanghai Medical College of Fudan University). For anesthesia, 1-mL pentobarbital (30 mg/kg) was administered intravenously. Bone marrow cells (8 mL) were obtained by aspiration from the tibia of each rabbit and divided into three 90-mm dishes in 8-mL Dulbecco’s Modified Eagle’s Medium. These brdU-labeled cells were centrifuged at 1500 rpm for 6 min. The supernatant was discarded and the cells were then individually suspended in culture medium, and incubated in a humidified 5% CO_2_ atmosphere at 37 °C. After culturing for four weeks, cells had proliferated to complete confluence. We defined adhesive cells as bMSCs. The cells were then harvested after treatment with 0.25% trypsin and 0.02% EDTA.

#### 3.1.2. Construction of the BMP-2 Eukaryotic Expression Vector and Packaging of the Recombinant Lentivirus

Rabbit cartilage tissue was isolated and ground in liquid nitrogen, and total RNA was extracted from the ground tissue using TRIzol reagent, in accordance with the manufacturer’s instructions. cDNA was prepared with an oligodT (18) primer, using Moloney murine leukemia virus (M-MLV) reverse transcriptase (Takara, Japan). The coding region of the BMP-2 gene (NM_001082650.1) was amplified by PCR from the cDNA template. The reaction conditions were as follows: 30 cycles of denaturation, annealing and extension (94 °C for 30 s, 56 °C for 30 s, and 72 °C for 60 s) and a final extension step at 72 °C for 5 min. EcoRI and BamHI restriction sites and protective bases were added to both ends of the PCR primers: EcoRI-BMP-2-F primer: 5′-GGAATTCATGGTGGCCGGGACC-3′, *Bam*HI-BMP-2-R primer: 5′-CGGGATCCCTAACGACACCCACAA-3′. The recombinant plasmid DNA was extracted and sequenced with primers that bound close to both ends of the multiple cloning site within the vector. 293TN cells (ATCC, USA) were transfected with the recombinant vector and the viral packaging plasmid mixture.

#### 3.1.3. Lentiviral Infection of bMSCs

The optimal multiplicity of infection (MOI) value for lentiviral infection of bMSCs was determined in a preliminary experiment. In brief, bMSCs were seeded into 96-well plates 24 h before viral infection. After seeding, cells were cultured under normal conditions for 24 h, and the standard virus solution (Lv-Control) was added to the culture media at various ratios. The cells were cultured under normal conditions for 72 h. GFP-Positive cells were observed using an inverted fluorescence microscope and the optimal MOI value was calculated. The infection efficiency was determined by fluorescence microscopy.

#### 3.1.4. MTT Assay

Seventy-two hours after infection, cells were trypsinized and prepared in a suspension, and the cell concentration was adjusted to 1 × 10^6^/mL. The cells were seeded into 96-well plates in a 100-μL total volume per well in triplicate. At 24, 48 and 72 h after incubation, 20-μL MTT (5 g/L) was added to each well, and the cells were cultured for an additional 4 h. The plates were vibrated for 10 min, and the absorbance (A) values at 490 nm were determined with a microplate reader to estimate changes in cell activity.

#### 3.1.5. Real-Time PCR

Total RNA was extracted from cells 72 h after viral infection using TRIzol reagent. cDNA was prepared from 2 μL RNA with oligodT (15) primer using M-MLV reverse transcriptase. Relative mRNA levels were detected by real-time PCR, using β-actin as an internal reference. The reaction conditions were as follows: 35 cycles of denaturation, annealing and extension (94 °C for 30 s, 56 °C for 30 s and 72 °C for 30 s) and a final extension at 72 °C for 10 min. The PCR primers were as follows: BMP2-F: 5′-CCTCCGGGGTATCACGCCTTTTA-3′, BMP2-R: 5′-GACACCCACAACCCTCCACAACCA-3′; β-actin-F: 5′-CCAAGGCCAACCGCGAGAAGATGA-3′, β-actin-R: 5′-GCAGCGCGTAGCCCTCG TAGATGG-3′. The lengths of PCR products were 238 base pairs (bp) and 180 bp, respectively. The results of relative quantification were analyzed by the ΔCt method, and the target gene expression level relative to that of the reference gene was expressed as 2^ΔCt^ = 2^Ctm−Ctn^.

#### 3.1.6. Western Blot Analysis

The cells were lysed in 0.5-mL cell lysis buffer for 30 min, and the supernatant was separated by centrifugation. Thirty μg of protein per sample was denatured by boiling and separated by 10% polyacrylamide gel electrophoresis and transferred to a polyvinylidene fluoride (PVDF) membrane. The membranes were blocked with Tris-buffered saline (TBS) containing 5% nonfat dry milk at room temperature, incubated with the appropriate primary antibody at a 1:500 dilution at 4 °C overnight, and incubated with the appropriate secondary antibody (horseradish peroxidase [HRP]-conjugated goat anti-mouse IgG) at a 1:5000 dilution at 37 °C for 1 h. The blots were visualized using an enhanced chemiluminescent (ECL) system by exposure after washing with TBST.

### 3.2. *In Vivo* Studies

#### 3.2.1. Animal Study Design

Thirty healthy, mature New Zealand white rabbits weighing an average of 3 kg (2.2–3.1 kg) were used. Animals were obtained from the Experimental Animal Department of Shanghai Medical College of Fudan University. The animal experiment was approved by the Animal Care and Use Committee of our college. The rabbits were randomly divided into three groups (10 per group): ACLs from the first group were reconstructed with a normal gastrocnemius tendon (control group), those from the second group were reconstructed with a gastrocnemius tendon wrapped by a total of 1 × 10^7^ bMSCs, which were infected with the control virus (Lv-Control) and immobilized in 0.2 mL fibrin glue (TISSEEL Kit, IMMUNO AG Vienna, Austria) (bMSCs+Lv-Control group), and those from the third group were reconstructed with a gastrocnemius tendon wrapped by a total of 1 × 10^7^ bMSCs, which were infected with the recombinant BMP-2 virus (Lv-BMP-2) and immobilized in 0.2-mL fibrin glue (bMSCs+Lv-BMP-2 group). Four and eight weeks post-operation, the animals were sacrificed for biomechanical and histological studies. Both knees of all rabbits were used for statistical analysis. All left knees of rabbits were used for mechanical testing, and all right ones of rabbits were used for histological examination.

#### 3.2.2. Surgical Procedure

ACL reconstruction surgery was performed under general anesthesia with 3% pentobarbital (30 mg/kg) using sterile techniques. A midline incision was made, and the gastrocnemius tendon was harvested. The knee joint was exposed by a lateral parapatellar arthrotomy. The native ACL was removed and tibial and femoral tunnels were created with a 2-mm drill. Both the treatment (bMSCs+Lv-Control group and bMSCs+Lv-BMP-2 group) and control grafts (gastrocnemius tendon) were coated with Tisseel fibrin glue immediately before insertion into the tunnels. On the treatment limbs, the autologous bMSCs+Lv-Control and bMSCs+Lv-BMP-2 were added to one of the micropipettes containing fibrin glue solution. The grafts were sutured to the periosteum using 2-0 Ethibond (Ethicon, Somerville, NJ, USA) on the femoral side. Subsequently, the tibial side was sutured with the knee in 10° of flexion and slight tensioning of the graft [31]. The wound was closed in layers. The animals were allowed to move freely in their cages after the operation.

#### 3.2.3. Mechanical Testing

Each rabbit from each group (*n* = 10) was sacrificed either 4 (*n* = 5) or 8 (*n* = 5) weeks post-operation for biomechanical evaluation. Limbs were stored at −20 °C until ready for testing. The limbs were thawed overnight at room temperature prior to use. The tibia and femur of the specimen were cut 4 cm from the joint. Mechanical tests were conducted using an Instron materials testing system machine (8874, Instron Co., Norwood, MA, USA). The joint was fixed by transverse pins between the U-shaped clamps with 30° of knee flexion and tilt of the femur and tibia. In this way, the pull force would pass through the axis of the graft. Pretension of the graft was performed by stretching the graft for 0.4 mm at a speed of 0.4 mm/s for a total of six times. Then, a tensile failure test was followed at a speed of 50 cm/s. The ultimate load and stiffness were measured using the software.

#### 3.2.4. Histological Examination

The knee joints of rabbits from each group (*n* = 10) were harvested either 4 (*n* = 5) or 8 (*n* = 5) weeks post-operatively. The gross shape of the grafts was observed and routine histological staining was then performed. After the animals had been sacrificed, the graft-bone complexes were fixed in 10% formalin for 48 h and then embedded and undecalcified in a methyl methacrylate compound. The samples were sectioned perpendicularly to the longitudinal axis of the femoral tunnel with a thickness of 5 μm using a microtome (SM2500, Leica, Nussloch, Germany). These sections were then stained with H & E for examination by light microscopy. Histological analyses were executed by two investigators that were blind to the animal surgery.

### 3.3. Statistical Analysis

Statistical analysis was performed using the SPSS for Windows release 13.0.0 statistical software (SPSS, Inc., Chicago, IL, USA). All data were statistically analyzed using ANOVA and are expressed as means ± standard deviations (SD). The mean values were compared using the paired sample *t*-test and the independent sample *t*-test. All *P* values were two-sided, and a *p* value < 0.05 was considered to indicate statistical significance.

## 4. Conclusions

The principal findings of this study demonstrated that the BMP-2-containing lentivirus infected efficiently bMSCs. Furthermore, tendons wrapped by BMP-2-transfected bMSCs had the potential to improve tendon–bone healing. We speculate the following explanations of the above results: Firstly, the proliferation of bMSCs and secretion of BMP-2, which targets cells by the autocrine or paracrine role, strengthens osteoinduction, and ultimately promotes tendon–bone healing. Secondly, bMSCs promote the integration of the tendon–bone interface by differentiating into bone and cartilage cells, or enhance collagen formation by differentiating into tendon cells, and eventually contribute to tendon–bone healing. However, the precise mechanism of action should be further examined.
